# Roles of Adrenergic α_1_ and Dopamine D_1_ and D_2_ Receptors in the Mediation of the Desynchronization Effects of Modafinil in a Mouse EEG Synchronization Model

**DOI:** 10.1371/journal.pone.0076102

**Published:** 2013-10-07

**Authors:** Chang-Rui Chen, Su-Rong Yang, Yuan-Yuan Liu, Wei-Min Qu, Yoshihiro Urade, Zhi-Li Huang

**Affiliations:** 1 Department of Pharmacology, Shanghai Medical College, Fudan University, Shanghai, China; 2 Institute of Brain Science, Fudan University, Shanghai, China; 3 Department of Molecular Behavioral Biology, Osaka Bioscience Institute, Suita, Japan; 4 State Key Laboratory of Medical Neurobiology, Shanghai Medical College, Fudan University, Shanghai, China; University G. D'Annunzio, Italy

## Abstract

**Background:**

Synchronized electroencephalogram (EEG) activity is observed in pathological stages of cognitive impairment and epilepsy. Modafinil, known to increase the release of catecholamines, is a potent wake-promoting agent, and has shown some abilities to desynchronize EEG,but its receptor mechanisms by which modafinil induces desynchoronization remain to be elucidated. Here we used a pharmacological EEG synchronization model to investigate the involvement of adrenergic α_1_ receptors (R, α_1_R) and dopamine (DA) D_1_ and D_2_ receptors (D_1_Rs and D_2_Rs) on modafinil-induced desynchronization in mice.

**Methodology/Principal Findings:**

Mice were treated with cholinergic receptor antagonist scopolamine and monoamine depletor reserpine to produce experimental EEG synchronization characterized by continuous large-amplitude synchronized activity, with prominent increased delta and decreased theta, alpha, and beta power density. The results showed that modafinil produced an EEG desynchronization in the model. This was characterized by a general decrease in amplitude of all the frequency bands between 0 and 20 Hz, a prominent reduction in delta power density, and an increase in theta power density. Adrenergic α_1_R antagonist terazosin (1 mg/kg, i.p.) completely antagonized the EEG desynchronization effects of modafinil at 90 mg/kg. However, DA D_1_R and D_2_R blockers partially attenuated the effects of modafinil. The modafinil-induced decrease in the amplitudes of the delta, theta, alpha, and beta waves and in delta power density were completely abolished by pretreatment with a combination of the D_1_R antagonist SCH 23390 (30 µg/kg) and the D_2_R antagonist raclopride (2 mg/kg, i.p.).

**Conclusions/Significance:**

These results suggest that modafinil-mediated desynchronization may be attributed to the activation of adrenergic α_1_R, and dopaminergic D_1_R and D_2_R in a model of EEG synchronization.

## Introduction

Agents that promote wakefulness have been employed as treatments for cognitive and behavioral symptoms of dementia for decades [1]–[3]. Modafinil [(2-[(diphenylmethyl) sulfinyl] acetamide)] is a potent, long-lasting wake-promoting substance [4]. It has been approved for use in the treatment of excessive daytime sleepiness [5], [6] and has been shown to efficaciously improve cognitive performance and to boost learning in methamphetamine-dependent participants [5], [7]–[9]. Recently, modafinil has also been used to treat cognitive impairments [10], with great safety and efficacy for cognitive enhancement of patient [11], [12].

It was reported that pharmacological blockage of cholinergic and noradrenergic activity provided a useful and valid model of the EEG synchronization. The model has been used to evaluate the desynchronization effects of the acetylcholinesterase inhibitor tacrine, which is used in the treatment of Alzheimer's Disease [13], [14]. In the present study, we used mice treated with cholinergic receptor antagonist scopolamine and monoamine depletor reserpine as a model of EEG synchronization, mimicking the nature and progression of pathological EEG synchronization to evaluate EEG desynchronization effects of modafinil.

To date, modafinil has only been shown to bind directly to the DA transporter and the NE transporter, but no apparent specific binding to other monoamine or neuropeptide receptors/transporters has been reported [15]. We hypothesized that modafinil may exert EEG desynchronization by acting on the noradrenergic and dopaminergic transmission system.

To identify the receptor involved in the EEG desynchronization by modafinil, we used the EEG synchronization model and adrenergic α_1_ receptor (R) antagonist terazosin, DA D_1_R antagonist SCH-23380, and D_2_R antagonist raclopride. The results indicated that modafinil decreased EEG synchronization via α_1_R, D_1_R, and D_2_R.

## Materials and Methods

### Animals

Male inbred C57BL/6J mice (weighing 20–28 g, 11–13 weeks old) were obtained from the Laboratory Animal Center, Chinese Academy of Sciences (Shanghai, China). The animals were housed individually at a constant temperature (24±0.5°C) with a relative humidity of 60±2% on an automatically controlled 12 h light/dark cycle (light on at 7:00 A.M.), and they had free access to food and water. The experimental protocols were approved by the Committee on the Ethics of Animal Experiments of the Fudan University Shanghai Medical College (Permit Number: 20110307-049). Additionally, all efforts were made to minimize animal suffering and to use only that number of animals necessary to produce reliable scientific data.

### Chemicals

Modafinil, adrenergic α_1_R antagonist terazosin, DA D_1_R antagonist SCH-23390, D_2_R antagonist raclopride, cholinergic receptor antagonist scopolamine hydrobromide and monoamine depletor reserpine were purchased from Sigma-Aldrich (Sigma-Aldrich, St. Louis, MO). All drugs were freshly prepared prior to use, and injection volume (10 ml/kg) was kept constant. The dosage selections, route of drug administration, and injection time of different compounds were based on preliminary experiments and pharmacokinetic considerations. Modafinil and reserpine were suspended and all other drugs were dissolved in saline containing 0.5% dimethylsulphoxide (DMSO).

### Surgery

Under chloralhydrate anesthesia (360 mg/kg, i.p.), mice were chronically implanted with electrodes for polysomnographic recordings of EEG and electromyogram as described earlier [4], [16]–[18]. Two stainless steel screws (1 mm in diameter) were inserted through the skull into the cortex (antero-posterior, +1.0 mm; left–right, −1.5 mm from bregma or lambda) according to the atlas of Franklin and Paxinos [19] and served as EEG electrodes. All electrodes were attached to a microconnector and fixed onto the skull with dental cement. The EEG recordings were carried out by means of a slip ring designed so that the behavioral movement of the mice would not be restricted. After a 10-day recovery period, the mice were housed individually in transparent barrels and habituated to the recording cable for 3–4 days before polygraphic recording.

### Electroencephalographic Recordings

Cortical EEG activity was recorded in awake, freely moving mice contained in a plexiglass cage. Lightweight cables were connected to the implanted electrodes and cortical activity was recorded differentially against the cerebella connection. The EEG signals were amplified and filtered (EEG, 0.5±30 Hz), then digitized at a sampling rate of 128 Hz, and recorded using SLEEPSIGN software as described before [20]. The raw EEG signal was displayed on one EEG channel. In addition, it was passed through software-controlled band-pass filters and the following frequency bands were separated and displayed individually on four additional channels: delta: 0.5–4 Hz; theta: 4–8 Hz; alpha: 8–12 Hz; beta: 12–20 Hz. The average peak-to-peak amplitude was computed for each frequency band by the fast Fouried transformation (FFT) method by SLEEPSIGN automatically. Fourier transformation was allowed calculation of the power variable (µV^2^). Absolute power spectra of EEG signals were computed every 10 s from 0–20 Hz in steps of 0.25 Hz. Since EEG signals in individual mice differ in their magnitude, absolute power spectra were transferred into relative changes, taking a synchronization value (mean for 30 min after scopolamine injection) as 100%, changes in each individual EEG band were analyzed in percents with respect to the synchronization value.

### Drug Treatment

The mice (5–9 in each group) were injected with reserpine (10 mg/kg) and approximately 10 h later, scopolamine hydrobromide (2 mg/kg). Subsequently, i.p. administering the following drugs to different mice pre-treated with reserpine and scopolamine: vehicle, cumulative doses of modafinil (22.5, 22.5, and 45 for a total final dose of 90 mg/kg) at 30 min intervals. Further groups of mice received combination treatment with terazosin (0.5, 1 mg/kg), raclopride (2 mg/kg), SCH-23390 (30 µg/kg) and modafinil 90 mg/kg. These mice received an initial injection of terazosin (0.5, 1 mg/kg), raclopride (2 mg/kg) and SCH-23390 (30 µg/kg, these doses were chosen because they did not produce significant effects by themselves), followed by modafinil 90 mg/kg or saline. Successive injections of each compound were given in 30 min intervals.

### Data Analysis

All data were expressed as the mean±SEM (n = 5–9). Statistical analysis was performed with SPSS 17.0 (SPSS Inc., Chicago, IL). The amplitude of delta, theta, alpha and beta was analyzed by one-way repeated measures analysis of variance (ANOVA) followed by the Fisher probable least-squares difference (PLSD) test to determine whether the difference among groups was statistically significant. The power density data were analyzed using the *t*-test. In all cases, *P*<0.05 was taken as the level of significance.

## Results

### Effects of modafinil on the amplitude at all frequency bands between 0 to 20 Hz

**Figure 1:**
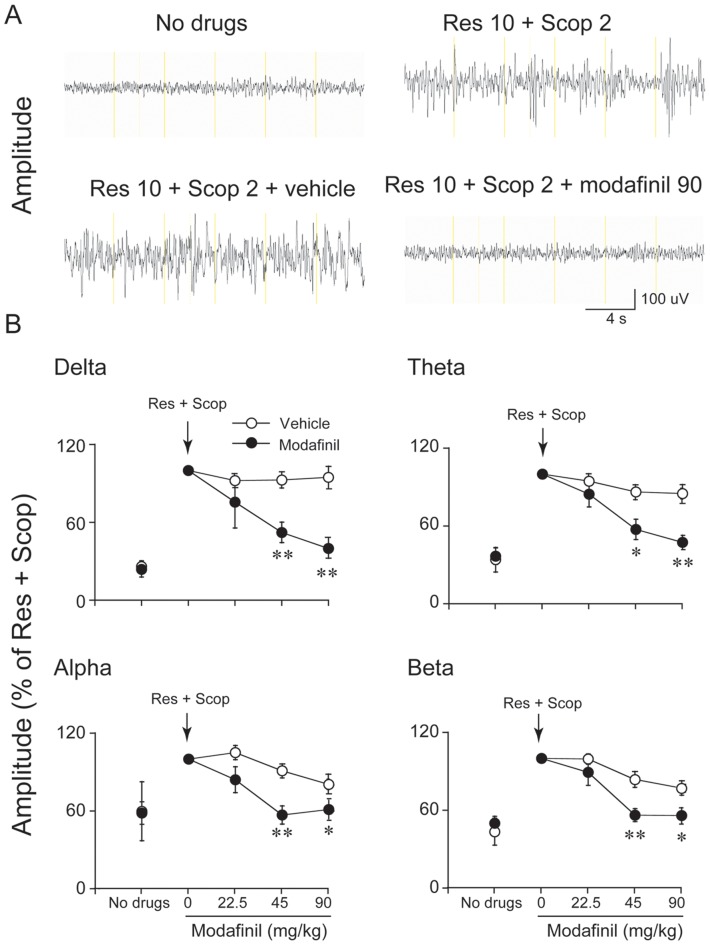
Modafinil decreased EEG amplitude in the synchronized model. (A) Examples of raw cortical EEG activity in untreated mice (no drug), after combined reserpine (Res; 10 mg/kg, i.p.) and scopolamine (Scop; 2 mg/kg, i.p.) treatment, and after additional injections of vehicle or modafinil (90 mg/kg, cumulative dose). (B) Time-course changes produced by the i.p. administration of modafinil. Res and Scop treatment increased amplitude in the delta (0–4 Hz), theta (4–8 Hz), alpha (8–12 Hz), and beta (12–20 Hz) bands relative to levels before drug administration. We took the value of amplitude in the delta, theta, alpha, and beta bands induced by Res and Scop as 100%. The independent open and filled circles indicate the baseline level. The connected open and filled circles represent vehicle or modafinil at 22.5, 45, 90 mg/kg. Modafinil (22.5–90 mg/kg, i.p.; administered in successive cumulative doses of 22.5, 22.5, 45 mg/kg) reversed increase in EEG amplitude produced by treatment with Res and Scop. The arrows indicate the times of drug injection. Values are means±SEM (n = 5–9). *P<0.05, **P<0.01, compared with vehicle control as assessed by one-way repeated ANOVA, followed by the PLSD testing.

To determine whether modafinil has any EEG desynchronization effects, we first injected reserpine and scopolamine into mice, then administered modafinil 30 min later. As shown in Figure 1A, the EEG showed desynchronized activity relative to the normal waking EEG. The EEG of mice shifted to continuous, large-amplitude synchronized activity after administration of a combination of reserpine (10 mg/kg) and scopolamine (2 mg/kg), as in previous works [13], [14]. Then, mice pre-treated with reserpine and scopolamine were i.p. re-injected with cumulative doses of modafinil (22.5, 22.5, and 45 for a total final dose of 90 mg/kg; n = 5–9; One-way ANOVA) at 30 min intervals. This 90 mg/kg dose of modafinil produced desynchronization (as compared to the effects of vehicle). This desynchronization was characterized by a general decrease in the amplitude of all frequency bands. Analysis of band-pass filtered activity showed that amplitude increased for all frequency bands between 0 to 20 Hz, but the increase was most pronounced in the lower frequency bands. When the amplitude after reserpine + scopolamine treatment was taken as baseline, the EEG amplitude of delta, theta, alpha, and beta activity increased 3.85-, 2.94-, 1.67-, 2.27-fold after reserpine and scopolamine injection, respectively (Figure 1B). Modafinil significantly suppressed the EEG amplitudes of delta, theta, alpha, and beta activity (delta: F_(1,10)_ = 18.76, *P*<0.01; theta: F_(1,10)_ = 12.17, *P*<0.01; alpha: F_(1,10)_ = 9.97, *P*<0.05 and beta: F_(1,10)_ = 21.3, *P*<0.01) relative to the vehicle treated group. There was no significant difference in EEG amplitude between modafinil 22.5 mg/kg and the vehicle. Modafinil at 45 mg/kg significantly decreased the EEG amplitude of delta, theta, alpha, and beta activity by 43.5%, 33.5%, 37.5%, 32.7%, respectively, relative to vehicle. When given at 90 mg/kg, modafinil significantly suppressed the EEG amplitude of delta, theta, alpha, and beta activity by 58%, 42.3%, 24.1%, and 27.2%, respectively. These results clearly indicate that modafinil exerts an EEG desynchronization effect.

### Effects of terazosin on the modafinil-induced decreased in amplitude

To determine whether the noradrenergic system is involved in the desynchronization effects of modafinil, mice were pretreated with terazosin, an antagonist of adrenergic α_1_R. In model mice given reserpine and scopolamine, injections of terazosin alone (1 mg/kg) produced no significant changes in EEG amplitude in any frequency band between 0–20 Hz (n = 5, data not shown).

**Figure 2:**
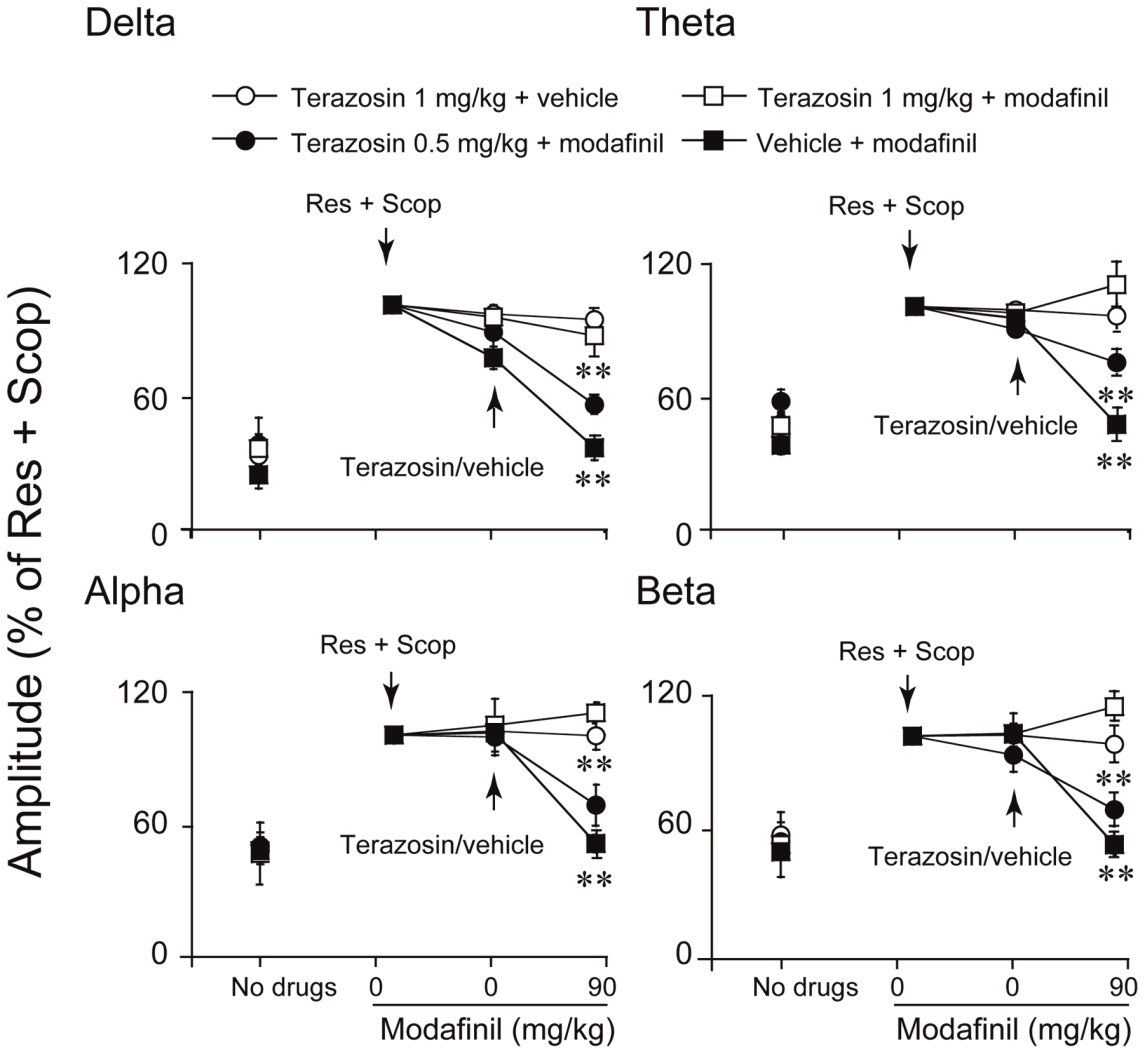
Pretreatment with terazosin (0.5, 1 mg/kg) reversed the decrease in EEG amplitude produced by modafinil (90 mg/kg, i.p.). The independent open and filled graphics indicate the baseline level. The connected open and filled graphics indicate different drug treatments (Res+Scop; subsequent vehicle or terazosin; subsequent, modafinil 90 mg/kg). We took the value of amplitude in the delta, theta, alpha, and beta bands induced by Res and Scop as 100%. The increase in EEG amplitude may be interpreted as synchronization and a decrease in EEG can be interpreted as a desynchronization. The arrows indicate the times of the drug injection. Values are means±SEM (n = 5–9). **P<0.01, compared with vehicle control as assessed by one-way repeated ANOVA, followed by PLSD testing.

Modafinil at a dose of 90 mg/kg induced the decrease in EEG amplitude of 0–20 Hz frequency band. The effect was found to be completely antagonized by terazosin 1 mg/kg relative to the vehicle control, as indicated by the fact that the synchronization of EEG amplitude in the delta, theta, alpha, and beta bands was almost identical to that of the vehicle control (n = 5, Figure 2). Terazosin at 0.5 mg/kg partly antagonized the effects of modafinil (n = 6, Figure 2). These results indicate that the EEG desynchronization caused by modafinil is mediated by adrenergic α_1_R.

### Effects of SCH-23390 and raclopride on modafinil-induced decreases in amplitude

**Figure 3:**
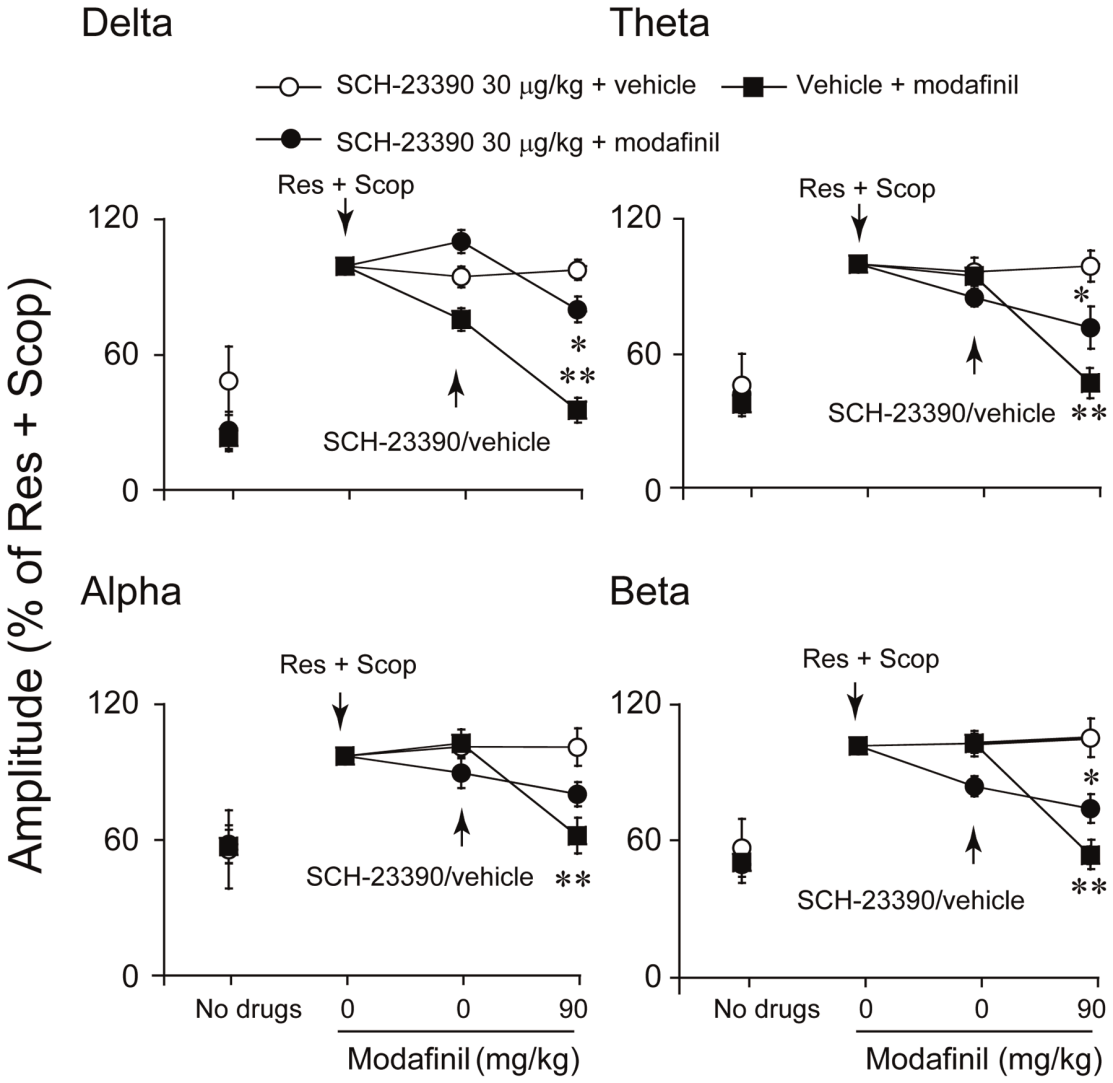
Pretreatment with SCH-23390 30 µg/kg antagonized the amplitude of alpha band increase induced by modafinil at 90 mg/kg. We took the value of amplitude in the delta, theta, alpha, and beta bands induced by Res and Scop to be 100%. The arrows indicate the times of drug injection. Values are means±SEM (n = 5–9). *P<0.05, compared with vehicle control as assessed by one-way ANOVA, followed by the PLSD test.

**Figure 4:**
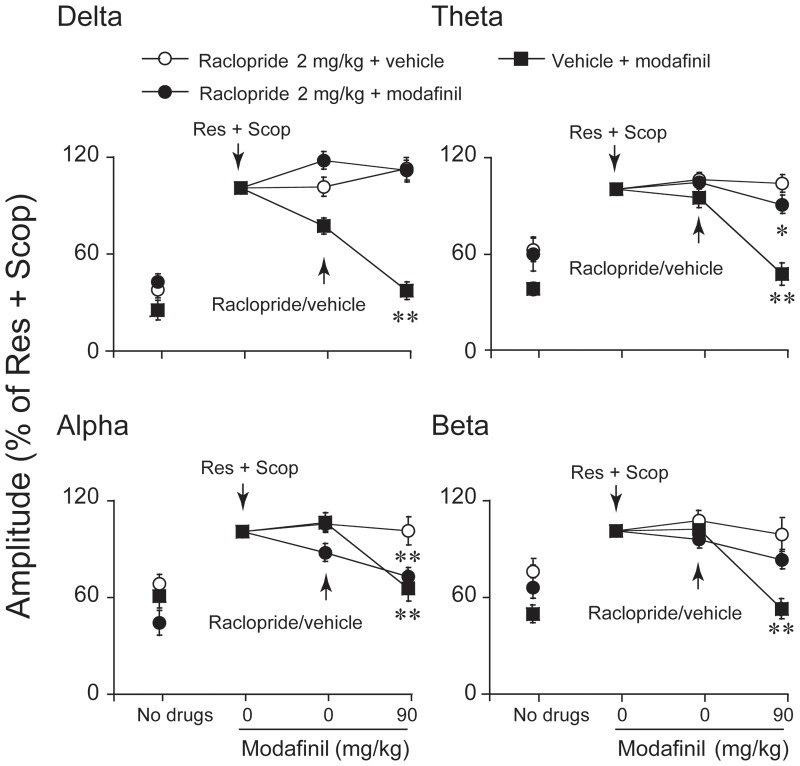
Pretreatment with raclopride blocked the increase in the amplitudes of the delta and beta bands induced by modafinil at 90/kg, but those in the alpha and theta frequency bands were not. We took the value of the amplitude in the delta, theta, alpha, and beta bands induced by Res and Scop to be 100%. The arrows indicate the times of drug injection. Values are means±SEM (n = 5–9). *P<0.05, **P<0.01, compared with vehicle control as assessed by one-way ANOVA, followed by the PLSD test.

**Figure 5:**
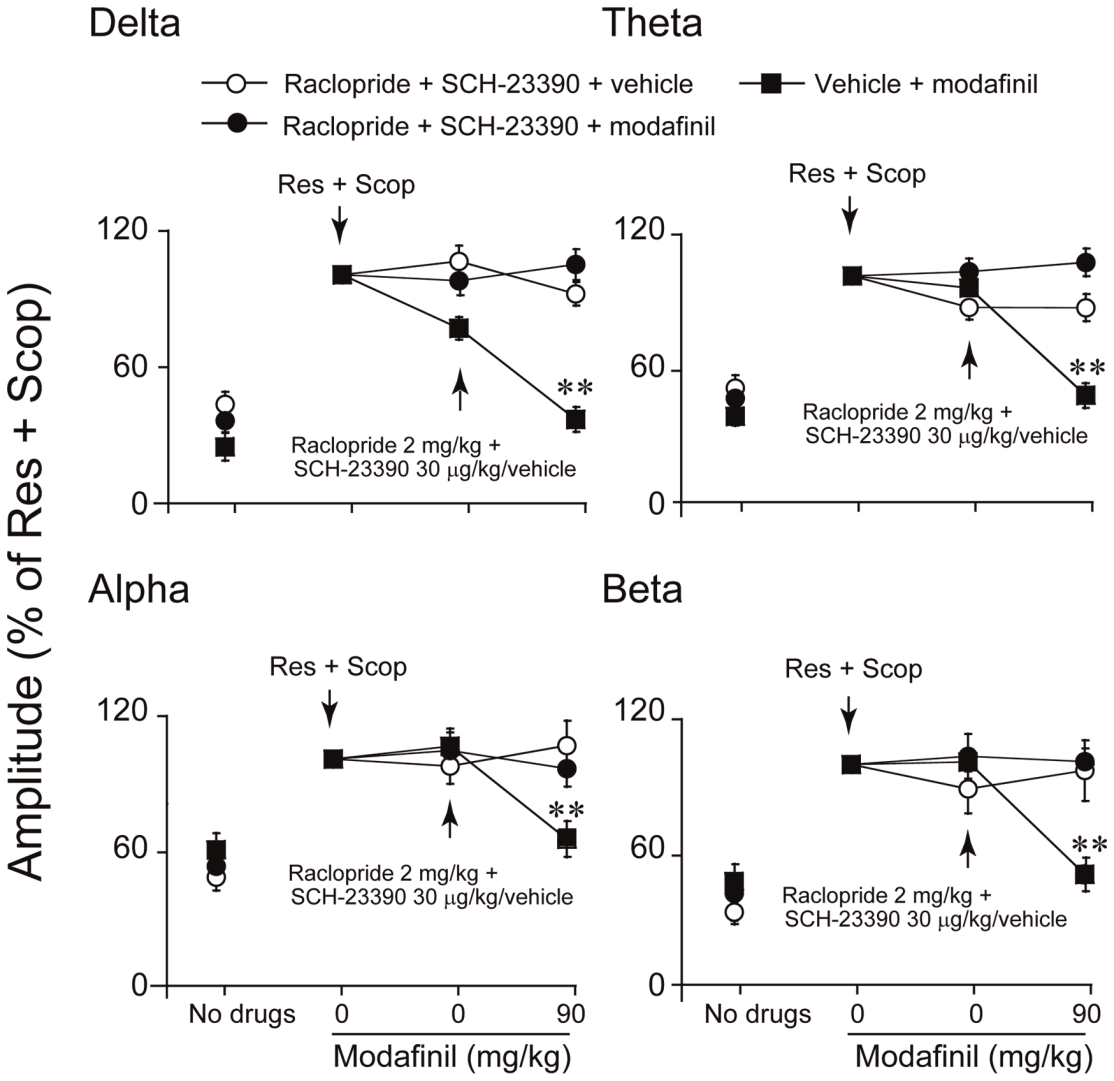
Combination treatments with SCH-23390 and raclopride completely blocked the decrease in the amplitudes of the delta, theta, alpha, and beta bands induced by modafinil at 90 mg/kg. We toke the value of amplitude in the delta, theta, alpha and beta bands induced by Res and Scop as 100%. The arrow indicate when the drug injection. Values are means±SEM (n = 5–7). Data assessed by one-way ANOVA, followed by the PLSD test.

To determine whether the dopaminergic system might be involved in the desynchronization effects of modafinil, modafinil-treated mice were pretreated with SCH-23390, an antagonist at D_1_R, or/and with the D_2_R antagonist raclopride. The decreased EEG amplitude at any frequency band between 0–20 Hz induced by modafinil at a dose of 90 mg/kg was found to be partly antagonized by SCH-23390 30 µg/kg (n = 5–7, Figure 3) or raclopride 2 mg/kg (n = 5–9, Figure 4). Modafinil at 90 mg/kg significantly decreased the EEG amplitude of delta activity by 17.8%, theta by 28.6%, and beta by 30.2%, but the EEG amplitude of alpha activity did not show any significant difference from that of SCH-23390 30 µg/kg pretreated mice given vehicle instead of modafinil ((n = 5–7, Figure 3). Modafinil at 90 mg/kg significantly decreased the EEG amplitude of theta and alpha activity by 12.7% and 28.4%, but the EEG amplitudes of the delta and beta bands did not show significant differences from those of mice pretreated with raclopride at 2 mg/kg and then given vehicle instead of modafinil (n = 5–9, Figure 4). SCH-23390 and raclopride combination treatment completely blocked the decrease in EEG amplitude induced by modafinil (n = 5–8, Figure 5).

### Effects of adrenergic α1R, D1R, and D2R antagonist on the changes in power density caused by modafinil

**Figure 6:**
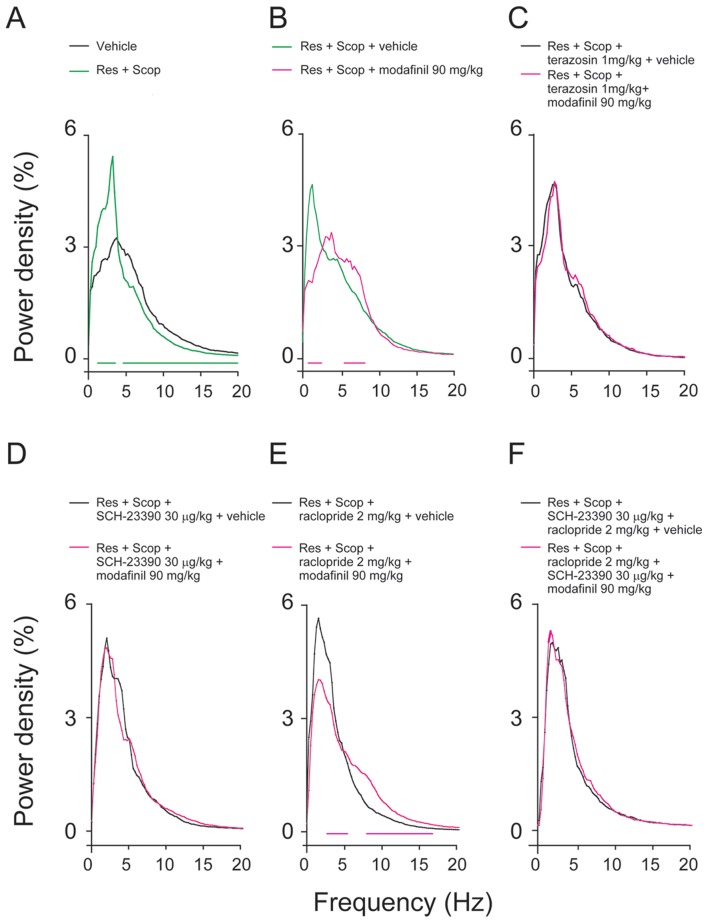
The EEG power density changed under different drug treatments. (A–B) The green, black, and red lines indicated changes in EEG power density when the different drug treatments (no drugs, Res+Scop, subsequent vehicle/modafinil 90 mg/kg) were present. The horizontal green and red lines at zero indicate significant changes. Modafinil decreased the power density of the delta band, and increased the intensity of the theta, alpha, and beta bands. The black lines indicate changes in EEG power density change when at (C) terazosin concentrations of 1 mg/kg, (D) SCH-23390 30 µg/ml, (E) raclopride 2 mg/kg, and (F) the two antagonists combination treatments. The red lines indicate that EEG power density changes when 90 mg/kg modafinil was administered after antagonist pretreatment. This decreased the power density of the delta band, and increased the intensity of the theta, alpha, and beta bands induced by modafinil was blocked by terazosin, SCH-23390 and combination SCH-23390 with raclopride. The power density of delta was still decreased after raclopride 2 mg/kg pretreatment. The frequency represents EEG spectral components between 0–20 Hz. The increase in EEG power is indicative of synchronization of EEG at the delta frequency and a decrease in delta power is indicative of desynchronization. The horizontal red lines at zero indicate significant changes. Values are means±SEM (n = 5–9). *P<0.05, **P<0.01, compared with vehicle control as assessed by one-way ANOVA, followed by PLSD testing.

As shown in Figure 6A–B, there were clear differences in EEG power densities between the mice treated with vehicle and those given reserpine and scopolamine combination treatment. Mice treated with reserpine and scopolamine showed significantly higher delta power density and lower theta, alpha, and beta power density (n = 5–9). Modafinil at 90 mg/kg decreased the delta power density and significantly increased theta power density (n = 5–9). In general, pretreatment with terazosin blocked changes in power density in all bands (0–20 Hz) (n = 5, Figure 6C). The decreased delta and increased theta power density induced by modafinil was antagonized when pretreated with terazosin 1 mg/kg. As shown in Figure 6D–E, the decrease in delta power density induced by modafinil was completely antagonized by SCH-23390 but only partially blocked by raclopride. The change in power density in all bands (0–20 Hz) induced by modafinil 90 mg/kg was completely blocked by a combination of SCH-23390 and raclopride (n = 5–8, Figure 6F). These data suggested that both D1R and D2R mediate the EEG desynchronization action of modafinil.

## Discussion

The present experiments showed that modafinil reversed the slowing of the EEG caused by the anticholinergic scopolamine and the monoamine depletor reserpine. The EEG desynchronization effect of modafinil is mediated by adrenergic α_1_ and DA D_1_ and D_2_ receptors.

Increases in the delta power spectra were here taken as indicative of synchronization while EEG activation was taken as indicative of desynchronization. Desynchronization consisted of blockage of the slow, high-amplitude waves in the present study [21]. Cortical neuronal activities are under the control of cholinergic, dopaminergic, and noradrenergic modulatory systems [22], [23]. Loss of cholinergic and monoaminergic inputs to the cortical mantle can result in slowing of the EEG and loss of desynchronization [13], [24]. It has been reported that increases in the amplitude of all three frequency bands is roughly the same in rats given either 1 or 5 mg/kg scopolamine [14]. In the present study, we used reserpine 10 mg/kg and scopolamine 2 mg/kg to produce a reliable loss of low-voltage, fast-wave activity in mice to mimic EEG synchronization. Our results showed that modafinil reversed the slowing of the EEG, decreased the power density of the delta power spectra, and increased the power density of higher-frequency waves, as in previous reports [25], [26]. The study raised the possibility that modafinil may be used to treat diseases with abnormally synchronized activity.

The importance of the role of EEG synchronization in modulating epileptiform abnormalities has also been observed in various forms of epilepsy [27]. High-frequency stimulation, such as deep brain stimulation has been found to significantly decrease generalized tonic-clonic seizures via EEG desynchronization in animals [28]. This has been successfully applied in therapy for epileptic patients [29], [30]. We have shown that modafinil exerts a dose-dependent antiepileptic effect mediated by adrenergic α_1_ and histaminergic H_1_ receptors but not by the adrenergic α_2_ receptor or dopaminergic D_1_ or D_2_ receptors in maximal electroshock and pentylenetetrazol kindling models [31]. In the model used in the present study, we did not find histaminergic H_1_ receptors to be involved in the desynchronization effects of modafinil (data not shown), suggesting that modafinil exerts EEG desynchronization mediated by different receptor mechanisms according to different models.

DA receptors are subdivided into D_1_-like (D_1_ and D_5_) and D_2_-like (D_2_, D_3_, and D_4_) receptors [32]. The D_1_R and D_2_R are the most abundantly expressed receptors for DA in the brain, whereas D_3_R, D_4_R, and D_5_R have lower abundance and a very restricted localization [33]. D_1_R and D_2_R play different roles in EEG changes caused by differences in modulation of dopaminergic transmission. D_1_R is a postsynaptic receptor, and administration of D_1_R agonist has been found to induce EEG desynchronization activity and behavioral arousal [34], [35]. D_2_R has two variants, a short form and a long form, representing presynaptic autoreceptors and postsynaptic receptors, respectively [36]. Because of this, the roles of D_2_R in EEG changes are relatively complex. Systemic administration of the D_2_R antagonist raclopride synchronizes EEG activities and increases EEG power density in low-frequency bands [26], [37], [38]. Dopaminergic agonists have been found to exert biphasic effects in the changes of EEG spectral power. Low doses reduce wakefulness and increase EEG power spectra, and large doses have the opposite effect, eliciting desynchronization of the EEG [26], [37], [39]. The administration of either low or high doses of D_2_R agonist to rats that have already been treated with D_1_R antagonist elicits a marked sedative response associated with EEG synchronization, and the effects can be prevented by D2R blockers [40]. This suggests that D_1_R plays an important role in the mediation of EEG desynchronization. D_2_R KO mice have been shown to exhibit a significant decrease in the power density of non-rapid-eye-movement sleep over the frequency range of the delta activity during the dark period [41]. However, pretreatment with D2R antagonist at doses that preferentially acts at presynaptic sites reversed the effects of low doses of D_2_R agonist [37]. This suggested that the EEG synchronization induced by D2R agonist was mainly mediated by presynaptic D_2_R.

Although modafinil is known to affect multiple neurotransmitter systems, such as catecholamines, serotonin, glutamate, GABA, orexin, and histamine, this drug increases extracellular levels of DA in the nucleus accumbens (NAc) and medial prefrontal cortex [42]. By using D_2_R knockout mice in combination with a DA D_1_R antagonist, we reported that both D_1_R and D_2_R are essential to the arousal effects of modafinil, with especially D_2_R being more important than D_1_R in these effects [4]. The results suggested that modafinil enhances extracellular levels of DA mainly by targeting at D_2_R. D_2_R and adenosine A_2A_R are colocalized in the NAc. Together with other recent data, we proposed that adenosine acting on excitatory A_2A_Rs can modulate the activity of GABAergic output neurons in the NAc to inhibit arousal and promote sleep. On the other hands, activation of the inhibitory D_2_R system suppresses the GABAergic neurons in the NAc to disinhibit the inhibitory actions to arousal systems and promote wakefulness [42], [43].

In the present study, desynchonization models by scopolamine and reserpine are different from synchronized pattern of non-rapid-eye-movement sleep. We found that D_1_R might be more important than D_2_R in the mediation of modafinil-induced EEG desynchronization. In addition, α1-adrenoceptors are also involved in the response.

Adrenergic signaling in the CNS plays a prominent role in the timing of sleep states and in the regulation of changes in EEG. α_1_-adrenoceptor agonist and α_2_-adrenoceptor antagonists, which increase noradrenergic transmission, induce a decrease in delta power and promote wakefulness [26]. A wealth of pharmacological data demonstrates the necessity of adrenergic receptors in the response to modafinil. Modafinil can bind to the NET [44], block the reuptake of NE [45], and enhance LC noradrenergic activity and transmission [46]–[49]. Although modafinil does not bind to α_1A_Rs, its effects on vigilance and the EEG change were tested using adrenergic α_1_R antagonist, showing that EEG desynchronization can be antagonized by α_1_R antagonists [15], [26], [50]. Central pharmacological blockage or genetic ablation of α_1B_R markedly attenuates the behavioral activation caused by modafinil [48]. At the physiological level, modafinil and the adrenergic α_1_R blocker prazosin desynchronize and synchronize the cortical EEG, respectively [26]. In the current study, we used adrenergic α_1_R blocker terazosin to prove that adrenergic α_1_R also mediate the desynchronization of modafinil.

In conclusion, modafinil exerts EEG desynchronization effects mediated by α_1_R, D_1_R, and D_2_R in a mouse model treated with reserpine and scopolamine.
